# Explainable multi-agent learning for adaptive terrorist network disruption

**DOI:** 10.1038/s41598-026-52996-5

**Published:** 2026-05-16

**Authors:** Vedat Dogan, Steven Prestwich, Barry O’Sullivan

**Affiliations:** Insight Research Ireland Centre for Data Analytics, School of Computer Science & IT, University College Cork, Cork, Ireland

**Keywords:** Explainable Multi-Agent Reinforcement Learning, Strategic Network Disruption, Decision Support Systems, Mathematics and computing, Physics

## Abstract

Disrupting terrorist networks remains a critical challenge for counter-crime units due to their adaptive, decentralized, and covert nature. Existing approaches provide valuable structural insights but often rely on static representations and do not capture the sequential and adversarial dynamics underlying real-world intervention processes. In this work, we propose an explainable, game-theoretic multi-agent reinforcement learning (MARL) framework for simulating and analyzing adaptive terrorist network disruption. We formulate the problem as a partially observable, sequential decision-making process between two agents: an *attacker*, representing a terrorist organization seeking to expand its operational or ideological influence, and a *defender*, modeling law enforcement entities tasked with disrupting influence and neutralizing key actors. The framework incorporates domain-informed reward functions that capture structural, behavioral, and resilience-related properties of networks, enabling both agents to learn policies through repeated interaction. A central contribution of this work is the integration of explainability into the learning framework, allowing the generation and quantitative evaluation of interpretable rationales for node-level intervention decisions. Rather than modeling the full complexity of radicalization processes, the proposed approach provides a stylized but tractable simulation environment for studying strategic interaction under uncertainty. Empirical evaluations on multiple real-world-inspired extremist network structures show that (i) disruption effectiveness improves with increasing intervention budget but exhibits non-monotonic and network-dependent behavior, (ii) outcomes are strongly shaped by attacker–defender strategy interactions rather than individual strategies alone, and (iii) learned policies produce consistent and structured explanation patterns that reveal underlying network vulnerabilities. These findings demonstrate that explainable MARL can provide actionable insights into adaptive intervention strategies and serve as a decision-support tool for intelligence-led policing in complex networked environments.

## Introduction

Terrorist networks pose persistent and evolving threats to global security by exploiting decentralized organizational structures, covert communication channels, and adaptive coordination mechanisms^1^. Comparative studies on structural vulnerability have shown that contact-based terrorist networks–particularly those observed in Mafia-type organizations^2^–tend to be more fragile than decentralized or ideology-driven terrorist networks under targeted interventions, yet often exhibit faster recovery dynamics following disruption^3^. Centrality-driven targeting strategies, defined as network intervention strategies in which nodes are selected for disruption based on their structural importance, further demonstrate that removing highly connected actors can substantially degrade operational capacity, although such effects may be transient if adaptive reconfiguration mechanisms are not accounted for^4^. These illicit systems are rarely static: they reorganize roles, redistribute influence, and reconfigure connections in response to external pressures, making sustained disruption a complex and dynamic challenge^5^. Reflecting this reality, the United Nations Office on Drugs and Crime (UNODC) has emphasized terrorist network disruption as a strategic priority, launching the Global Programme on Criminal Network Disruption (GPCD)^6^ in 2022 to support member states with analytical tools and operational frameworks for identifying and dismantling high-impact network structures. Central to these efforts is criminal network analysis, which seeks to identify structural vulnerabilities and critical actors whose removal or neutralization may best degrade network functionality.

Early and influential work in this domain established the utility of social network analysis (SNA) for law enforcement, highlighting concepts such as actor substitutability and role uniqueness in identifying optimal intervention targets^7^. Subsequent studies demonstrated that network topology strongly shapes vulnerability to disruption, with hub-targeted or bridge-node removals proving effective depending on whether networks exhibit scale-free, small-world, and hybrid structures^8^. Building on this foundation, individual-level metrics such as key player identification for fragmentation and reach (KPP1/KPP2) enabled more targeted and iterative intervention strategies^9^. Here, KPP1 identifies sets of actors whose removal maximally fragments the network, while KPP2 selects actors that collectively maximize reach across the network. Empirical investigations further revealed that centralized leadership structures tend to be more vulnerable, whereas flatter or compartmentalized organizations often display greater resilience to disruption^10–12^.

Beyond graph-theoretic and SNA-based approaches, related research has also examined crime through agent-based, game-theoretic, and statistical-physics perspectives. In particular, Perc et al. modeled recurrent crime as an emergent collective phenomenon in a spatial inspection game, showing how cyclic dynamics among criminals, inspectors, and ordinary individuals can arise endogenously rather than through fixed one-step assumptions^13^. D’Orsogna and Perc further reviewed a broad range of quantitative crime models, including hotspot models, self-exciting processes, agent-based approaches, adversarial evolutionary games, and network-based perspectives, thereby highlighting the value of dynamic and interaction-driven models for understanding criminal activity^14^. These studies are important because they emphasize that crime and enforcement are shaped by feedback, adaptation, and collective dynamics, rather than by static structures alone.

More recently, machine learning methods have been applied to criminal and terrorist networks, including approaches for uncovering hidden ties, predicting network evolution, and learning representations of illicit structures^15^. In particular, Lopes et al. showed that graph representation learning combined with machine learning can recover missing criminal partnerships, distinguish between different types of ties, and anticipate future criminal associations in evolving corruption networks^16^. Ribeiro et al. extended this direction using deep learning based on the GraphSAGE framework^17^, reporting strong performance for recovering criminal partnerships, predicting edge properties, and anticipating future associations and recidivism-related patterns in criminal networks^18^. While these studies demonstrate the potential of data-driven methods for criminal network analysis, they primarily focus on prediction, reconstruction, or representation learning rather than sequential intervention under strategic adversarial adaptation. Despite these advances, a growing body of evidence shows that terrorist networks actively adapt to enforcement actions by forming hidden ties, redistributing responsibilities, and shifting operations across organizational or geographic boundaries^19–21^. In several documented cases, interventions have triggered rapid reconfiguration or even counterintuitive increases in cohesion and efficiency following node removal^22–25^. Simulation-based and longitudinal studies have highlighted the importance of sequential, rather than simultaneous, interventions–particularly those targeting intermediaries, financial actors, or mediators–but also underscore the difficulty of anticipating downstream effects in adaptive systems^26–29^. More recent work has introduced domain-specific metrics for covert communication networks^30^, predictive models for uncovering hidden ties^15^, and resilience-oriented indicators such as centralization for detecting escalation phases^31–33^.

Collectively, this literature suggests that static intervention strategies are insufficient in adversarial settings characterized by feedback, learning, and temporal dependency^34^. A critical limitation shared by most existing approaches is their reliance on static or weakly dynamic representations of terrorist networks. While graph-theoretic methods provide valuable structural insights, they typically assume fixed topologies and passive responses to intervention^35,36^. As a result, they struggle to capture strategic co-adaptation, information asymmetry, and the sequential decision-making processes that govern real-world disruption efforts. In practice, defenders operate under partial observability due to incomplete intelligence, hidden relationships, and delayed or noisy information about network structure and individual behavior. At the same time, terrorist actors strategically respond to enforcement actions by altering behavior, recruitment, and connectivity over time, leading to adaptive and non-stationary network dynamics^19^. These interactions give rise to non-stationary dynamics and delayed consequences, where early interventions may prevent influence cascades, whereas delayed or misaligned actions can enable adversarial entrenchment.

In this study, we address these challenges by proposing a principled multi-agent reinforcement learning (MARL) framework for simulating and analyzing terrorist network disruption as a sequential, adversarial decision-making process. We model the interaction between two agents: an attacker, representing a terrorist organization seeking to expand or preserve its influence, and a defender, representing law enforcement or counter-crime units tasked with disrupting network influence propagation. The agents interact over a partially observable, evolving graph that encodes social, communicative, and functional dependencies among individuals, where each agent observes only a subset of the network structure and node attributes, reflecting incomplete and uncertain intelligence about relationships and individual behavior. A key novelty of our framework is the explicit incorporation of explainability into the decision-making process of both agents. In this context, explainability refers to the ability to associate each intervention action with structured, interpretable rationales grounded in network features, such as node centrality, bridge roles, and risk-related attributes. Unlike conventional post-hoc explanation techniques that approximate the behavior of black-box models, the proposed framework produces explanations that are directly aligned with the underlying decision process, enabling step-wise interpretation of strategic decisions, target selection, and adaptation dynamics. This capability is particularly important in high-stakes domains such as counter-terrorism, where transparent and interpretable decision support is essential for analyst trust and operational adoption, and remains largely underexplored in prior MARL-based approaches to network disruption, which typically rely on opaque policies^37^. This framework is designed to capture key characteristics of real-world disruption processes, including non-stationarity arising from adversarial co-learning, temporal dependencies in action outcomes, and the need to balance multiple, often competing objectives such as fragmentation, influence containment, and operational integrity. Within this setting, policies are learned through repeated interaction with the environment, allowing strategic behaviors to emerge endogenously rather than being imposed through fixed heuristics.

This learning-based perspective bridges the gap between static network interdiction models and adaptive, long-run approaches to terrorist network disruption. Beyond performance evaluation, we emphasize interpretability by analyzing emergent strategies and network evolution patterns, with the aim of generating actionable insights that may inform policy design and operational planning in complex, uncertain environments.

**Fig. 1:**
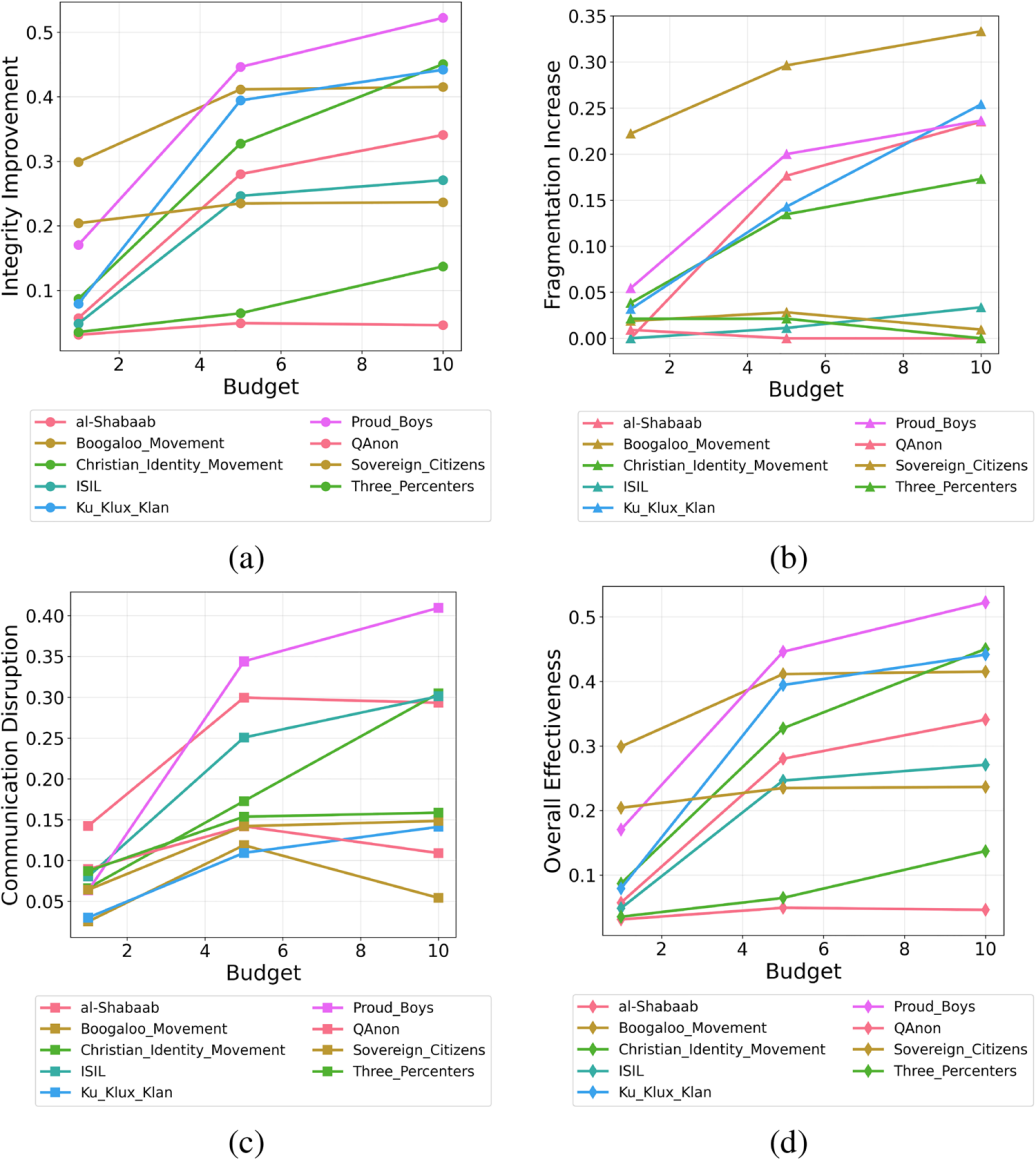
Budget-dependent disruption performance across extremist group networks. Disruption outcomes are reported as a function of defender budget using four complementary metrics: (**a**) network integrity improvement, (**b**) fragmentation increase, (**c**) communication disruption, and (**d**) overall effectiveness. Each curve corresponds to a different extremist network and values are averaged across evaluation episodes. Higher values indicate stronger disruption effects, although scales differ across subplots. The key observation is that marginal gains diminish at higher budgets for several networks, indicating that structural properties mediate how effectively additional resources translate into disruption.

## Results

This section reports the empirical performance of the proposed explainable multi-agent reinforcement learning framework across multiple extremist group networks. Results are organized around four dimensions: (i) budget-dependent disruption performance, (ii) attacker–defender strategy interactions, (iii) structural targeting and network evolution patterns, and (iv) explainability characteristics of agent decisions. All reported values are averaged across evaluation episodes for each group and budget configuration.

### Budget-dependent disruption performance

Figure 1 summarizes disruption performance as a function of defender budget across nine extremist group networks, evaluated using four complementary metrics: network integrity improvement, fragmentation increase, communication disruption, and overall effectiveness. Across all metrics, increased budget availability is generally associated with improved disruption outcomes; however, the magnitude and shape of these improvements vary substantially across groups.

Network integrity improvement in Fig. 1(a) exhibits rapid gains at lower budgets for several groups, followed by justifying returns at higher budget levels. Groups such as Proud Boys and Ku Klux Klan show pronounced integrity improvements as budget increases, whereas others, including QAnon and Three Percenters, display more modest responses. Fragmentation increase in Fig. 1(b) shows greater heterogeneity, with some networks exhibiting substantial increases in connected components and others remaining comparatively resistant even under higher budgets, indicating topology-dependent fragmentation behavior.

Communication disruption in Fig. 1(c) generally increases with budget but exhibits non-monotonic trends for certain groups, where intermediate budgets achieve disruption levels comparable to or exceeding those at maximal budgets. The aggregated overall effectiveness metric Fig. 1(d) reflects these combined dynamics, showing consistent but uneven gains across networks. Together, these results indicate that budget escalation alone does not yield uniform disruption benefits and that structural properties strongly mediate intervention outcomes.

Beyond aggregate improvements, Fig. 1 reveals systematic non-monotonicity in budget effectiveness. For several networks, the largest marginal gains occur between low and medium budget levels, after which additional resources yield diminishing improvements across multiple metrics. In contrast, a subset of networks exhibits delayed responsiveness, where structural and communication disruption only become pronounced at higher budgets. This divergence indicates that budget efficiency is strongly mediated by network topology, with certain structures enabling early leverage points while others require sustained intervention pressure before measurable degeneration emerges.

**Fig. 2:**
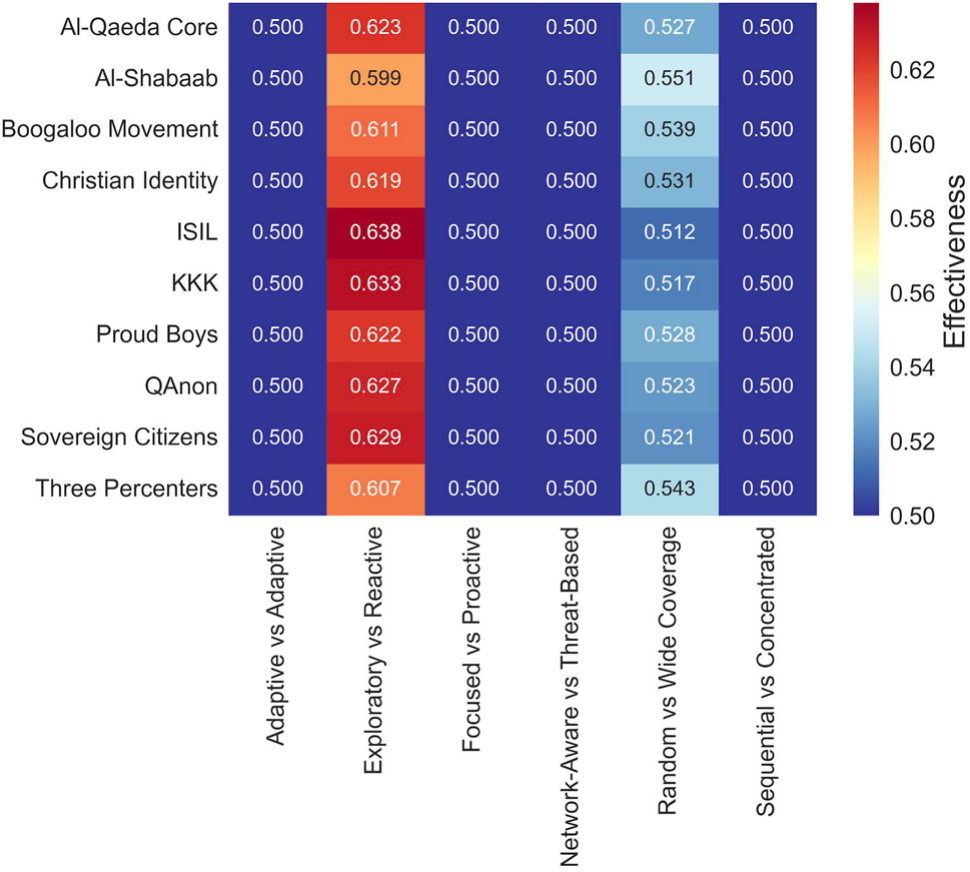
Effectiveness heatmap for attacker–defender strategy combinations. Each cell represents the overall disruption effectiveness achieved by a specific attacker–defender strategy pairing for a given extremist network. Values are normalized, with scores around 0.50 indicating no systematic advantage for either agent. The main finding is that disruption effectiveness is highly sensitive to strategy interactions rather than individual strategies alone, with certain pairings (e.g., exploratory vs. reactive) consistently outperforming others. This highlights the importance of adaptive and context-aware defense strategies in adversarial environments.

**Fig. 3:**
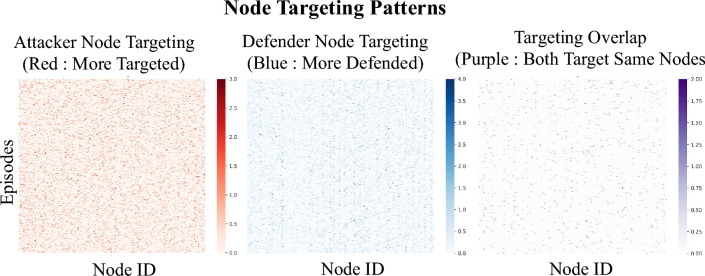
Node-level targeting frequency and attacker–defender overlap. Heatmaps show the normalized selection frequency of nodes across evaluation episodes for (**a**) the attacker, (**b**) the defender, and (**c**) their overlap. Color intensity represents the probability of a node being selected, aggregated and normalized over multiple runs. The key observation is that attacker strategies exhibit broader, exploratory targeting, whereas defender strategies concentrate on a smaller subset of structurally important nodes. The limited overlap between (**a**) and (**b**) indicates that defenders do not simply react to attacker choices but instead learn distinct, structure-driven targeting policies.

**Fig. 4:**
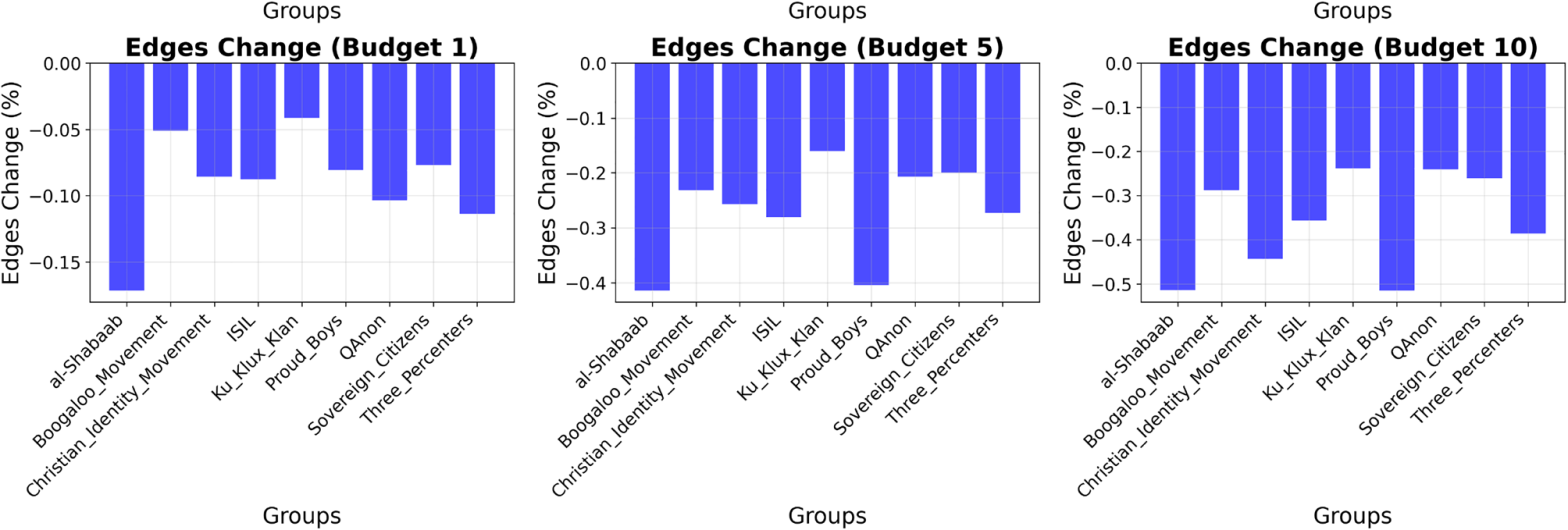
Network edge reduction under increasing intervention budgets. The proportion of remaining edges is shown for each extremist network at different defender budget levels (Budget 1, Budget 5, and Budget 10). Lower values indicate greater structural degradation of the network. While edge reduction generally increases with budget, the rate of decline varies substantially across networks. The main takeaway is that structural resilience differs across groups, with some networks requiring significantly higher intervention levels before substantial degradation occurs.

### Attacker–defender strategy interactions

Figure 2 presents a heatmap of overall effectiveness scores for attacker–defender strategy combinations across extremist group networks. Substantial variation is observed across strategy pairings, highlighting that disruption performance depends critically on the interaction between attacker and defender behaviors rather than on either strategy in isolation.

The “Exploratory vs. Reactive” pairing consistently achieves the highest effectiveness values across most groups, with scores exceeding 0.62 for networks such as ISIL and the Ku Klux Klan. In contrast, pairings such as “Focused vs. Proactive” and “Network-Aware vs. Threat-Based” cluster near the neutral baseline of 0.50, corresponding to no net advantage for either agent under these configurations. Notably, strategy effectiveness varies across groups even for identical pairings, underscoring the influence of underlying network structure on adversarial outcomes.

A notable pattern emerging from Fig. 2 is the asymmetric sensitivity of defender performance to attacker behavior. While several defender strategies achieve comparable outcomes against structured attacker policies, exploratory attacker behavior consistently challenges reactive defense, yielding elevated effectiveness scores across multiple networks. Conversely, proactive and threat-based defender strategies tend to compress differences among attacker policies, resulting in near-baseline effectiveness. This indicates that strategic uncertainty introduced by exploration plays a central role in shaping adversarial outcomes, particularly in networks with heterogeneous connectivity patterns.

### Node-level targeting patterns and structural overlap

Figure 3 presents node-level targeting behavior across evaluation episodes for both agents. The heatmaps visualize the normalized selection frequency with which each node is chosen at each episode, averaged across runs and stochastic action realizations. As a result, color intensities represent probabilistic targeting tendencies rather than raw episode counts, and fractional values naturally arise from normalization and averaging over multiple trajectories.

Attacker targeting patterns in Fig. 3(a) exhibit a broadly dispersed distribution, reflecting exploratory behavior and adaptive selection across the network. Despite this apparent visual uniformity, consistent vertical bands of higher intensity indicate nodes that are repeatedly selected across many episodes, suggesting stable strategic preferences rather than random action selection. Defender targeting patterns in Fig. 3(b), by contrast, show a more concentrated focus on a smaller subset of nodes, consistent with a learned emphasis on structurally influential or high-impact positions within the network.

The overlap heatmap in Fig. 3(c) highlights nodes that are concurrently targeted by both agents. Overlap regions are sparse, indicating that defender policies do not simply mirror attacker choices but instead prioritize different nodes based on learned assessments of downstream network impact. This limited overlap suggests a strategic divergence in targeting objectives, with defenders emphasizing structural leverage rather than reactive interception.

Importantly, these targeting patterns remain stable across episodes, pointing to policy-driven behavior shaped by adversarial learning dynamics rather than stochastic variability. The figure therefore illustrates how attacker and defender agents allocate attention across the network in a temporally consistent yet strategically distinct manner, revealing structurally salient nodes through repeated, normalized selection rather than isolated high-frequency events.

### Structural evolution under intervention

Figure 4 examines changes in network edge structure across increasing budget levels. At low budgets (Budget 1), edge removal is limited and uneven across groups. As budgets increase (Budgets 5 and 10), edge reduction becomes more pronounced and consistent across networks, indicating escalating structural degradation.

The magnitude of edge reduction varies by group, with some networks exhibiting steep declines in connectivity and others showing more gradual changes. These results align with earlier observations of heterogeneous fragmentation behavior and further demonstrate that structural resilience differs substantially across network types. The increasing uniformity of edge reduction across groups at higher budgets suggests a convergence toward common structural failure modes, despite initial heterogeneity in network organization.

### Explainability patterns and rationale diversity

**Fig. 5:**
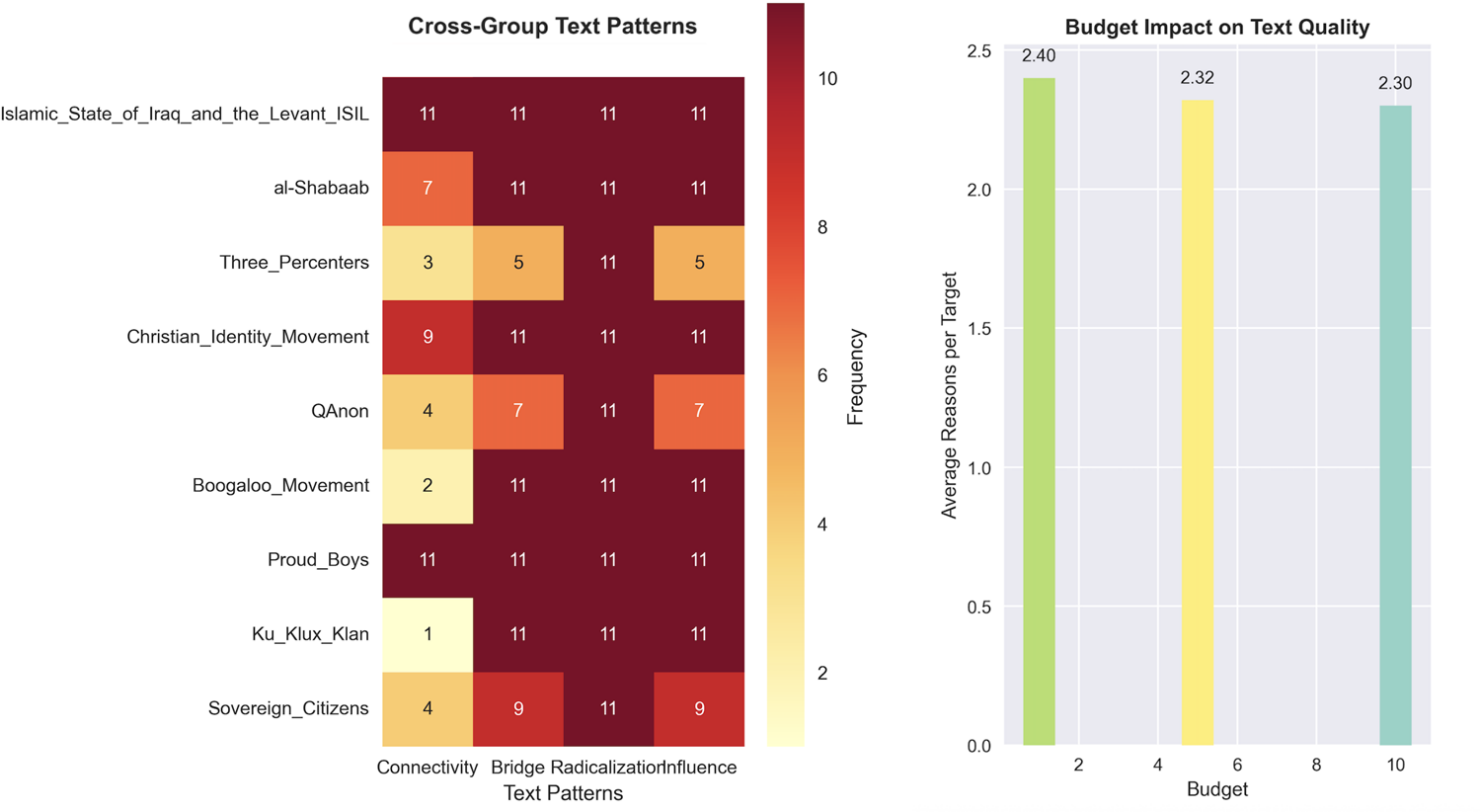
Distribution of explanation rationale types across extremist networks. Bars represent the relative frequency of explanation components generated by the framework, including connectivity, bridge roles, radicalization, and influence. While all explanation categories are present across networks, their relative importance varies, indicating that agent decisions are context-dependent. The key insight is that explanations are not random but systematically reflect underlying structural and behavioral characteristics of each network.

Figure 5 analyzes the frequency of explanation components generated by the framework across extremist groups. Four dominant rationale types–connectivity, bridge role, radicalization, and influence–appear consistently across groups, though their relative frequencies vary. Certain groups exhibit strong emphasis on bridge and influence-based explanations, while others show more balanced distributions across rationale types.

**Fig. 6:**
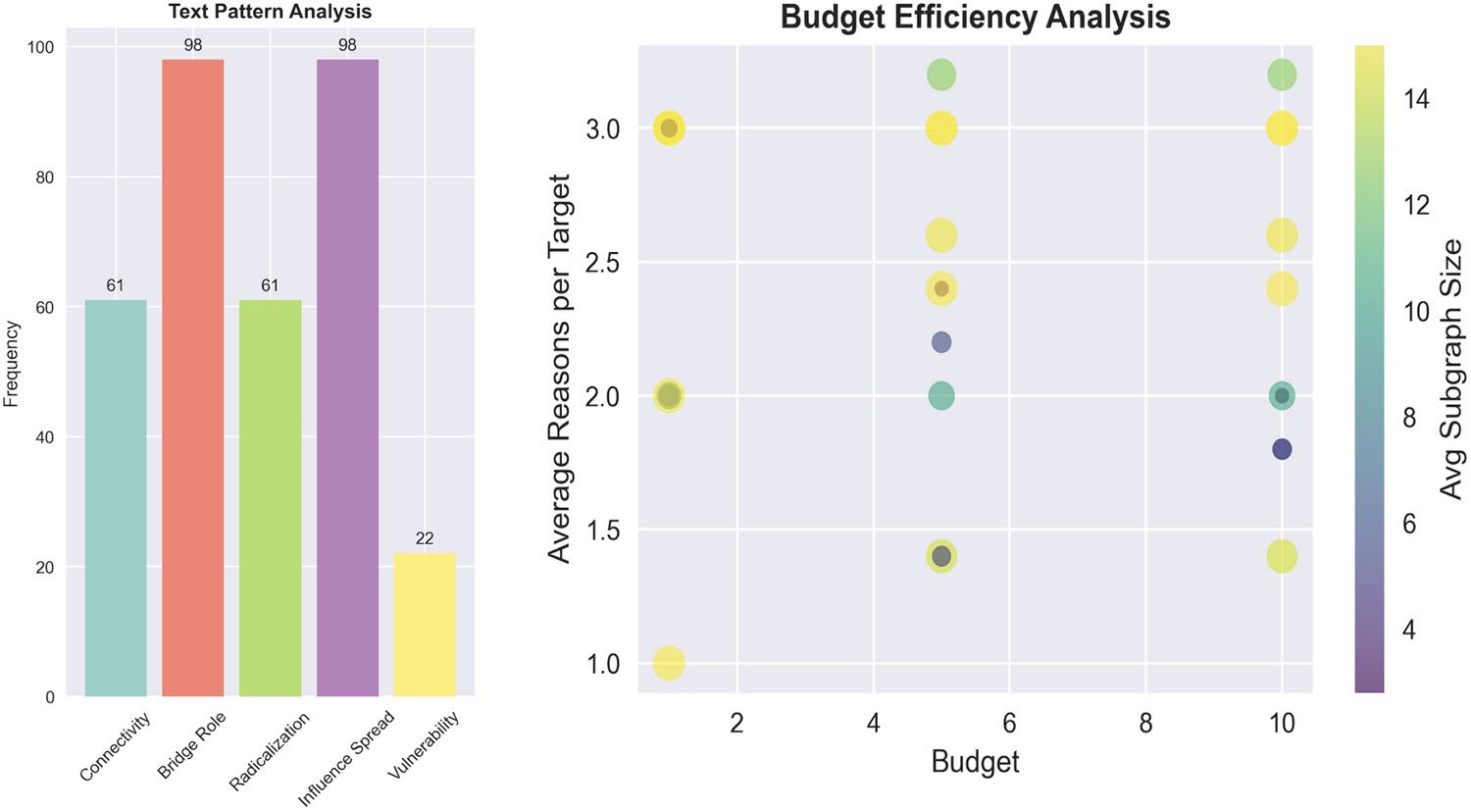
Explainability richness under varying budget and structural context. Bubble size represents the average number of distinct rationale components per explanation, plotted against defender budget and average subgraph size. Higher budgets are associated with larger bubbles, reflecting more detailed and diverse explanations. The main observation is that increased resource availability leads to richer, more context-aware reasoning rather than increased randomness, suggesting that explainability scales with both budget and structural complexity.

Figure 6 further examines explainability characteristics as a function of budget. At higher budgets, agents generate longer explanations with a greater number of distinct rationale components per action, indicating increased decision complexity rather than instability. The bubble plot reveals that explanation richness scales with both budget and average subgraph size, suggesting that agents incorporate broader contextual information when resources allow.

Taken together, Figures 5 and 6 indicate that explanation generation scales not only with resource availability but also with structural context. Higher budgets enable agents to reference a broader set of network attributes, resulting in explanations that are both longer and more diverse. Notably, this increase in explanation richness does not correspond to increased randomness; instead, rationale types recur systematically across groups, suggesting that the explainability mechanism captures stable, strategy-relevant features rather than producing ad hoc justifications. Collectively, these explainability results demonstrate that the framework produces structured, interpretable rationales that evolve systematically with budget and network context, providing transparent insight into agent decision-making processes.

## Discussion

This study introduced an explainable, multi-agent reinforcement learning (MARL) framework for terrorist network disruption, formalizing attacker–defender interaction as a sequential, partially observable stochastic game over evolving influence graphs, which are directed networks in which nodes represent actors (e.g., individuals, groups, or entities), and edges encode the capacity of one actor to affect the beliefs, behavior, decisions, or state of another over time. The empirical evaluation across multiple real-world-inspired extremist group networks indicates that (i) disruption outcomes improve with increased intervention budgets but with non-monotonic and group-dependent returns, (ii) effectiveness is strongly shaped by attacker–defender strategy interactions rather than by either policy class alone, and (iii) the proposed explainability components provide interpretable evidence of how agents allocate attention and how disruption patterns emerge over time and across budget regimes.

A consistent empirical pattern is that higher budgets tend to increase disruption performance across the reported metrics (network integrity improvement, fragmentation, communication disruption, and overall effectiveness), but the gain curves are not uniform across networks as presented in Fig. 1. In several groups, performance exhibits rapid improvement at lower budgets followed by saturation, suggesting that a subset of high-leverage interventions can account for much of the attainable benefit, while additional resources yield diminishing marginal impact. Conversely, some networks show comparatively muted sensitivity to budget escalation, consistent with more decentralized, compartmentalized, or redundancy-rich structures. These observations support the view that resource allocation alone does not determine disruption success: network topology and adaptation mechanisms mediate how interventions translate into structural and functional degradation.

Beyond budget, the cross-strategy evaluation highlights that disruption performance depends critically on the interaction between attacker and defender behaviors. The heatmap of attacker–defender combinations shows that certain pairings systematically yield higher effectiveness across multiple groups, whereas several combinations concentrate near a neutral baseline. This indicates that some strategy pairs approach a functional equilibrium where neither side consistently gains advantage, while other pairings create exploitable asymmetries. Importantly, the presence of group-dependent outcomes under the same strategy pairing suggests that strategic advantage is conditional on network structure and the distribution of node attributes. From a practical standpoint, this implies that fixed, one-size-fits-all intervention doctrines may underperform in heterogeneous operational settings; instead, adaptive selection of defensive strategy may be necessary to account for both observed adversary behavior and structural properties of the target network.

A central contribution of this work is the integration of explainability into both decision-making and post hoc analysis. Node-level action heatmaps provide localized evidence of which individuals are repeatedly targeted by attackers, defended by defenders, and mutually contested, enabling analysts to inspect whether policies concentrate on hubs, bridges, or structurally peripheral nodes. In parallel, budget-stratified summaries of edge reduction provide a global view of structural impact and show how resource availability scales the magnitude of disruption. The natural-language rationales further contextualize node selections by linking actions to interpretable indicators (e.g., bridge role, high centrality ranking, or elevated risk-related features). Together, these elements move beyond black-box performance reporting by offering traceable, analyst-facing explanations of *why* particular nodes are selected and *how* disruption patterns evolve. In high-stakes counter-crime contexts, such transparency is essential for auditability and for aligning algorithmic recommendations with operational constraints and accountability requirements.

The results suggest several implications relevant to intelligence-led policing and counter-crime planning. First, budget sensitivity varies substantially by group, implying that resource allocation should be guided by structure-aware assessments rather than uniform deployment rules. Second, strategy interaction effects indicate that defenders may benefit from selecting tactics that are robust to adversary probing and that can adapt as attacker behavior shifts. Third, the explainability layer provides a mechanism for translating learned policies into operationally meaningful narratives (e.g., “bridge isolation” vs. “hub neutralization”), supporting the integration of algorithmic recommendations into analyst workflows. While the present study is positioned as a simulation and decision-support framework rather than a prescriptive operational tool, these findings motivate the use of learning-based, interpretable models for exploring counterfactual intervention scenarios under uncertainty.

### Strengths and limitations

This study presents several strengths in both methodological design and empirical analysis. First, the proposed framework formulates terrorist network disruption as a sequential, adversarial decision-making problem, explicitly modeling the interaction between attacker and defender agents. This extends beyond traditional static or one-shot intervention approaches by capturing strategic co-adaptation and temporal dependencies in network evolution. Second, the integration of explainability into the multi-agent reinforcement learning framework represents a key contribution. By associating agent actions with structured, interpretable rationales derived from network features, the model provides transparency into decision-making processes, enabling analyst-facing interpretation of intervention strategies. This is particularly important in high-stakes domains such as counter-terrorism, where trust, accountability, and interpretability are essential. Third, the framework incorporates domain-informed reward functions that capture structural, behavioral, and resilience-related aspects of network disruption. This allows the model to evaluate interventions along multiple dimensions, including fragmentation, influence containment, and network integrity, rather than relying on a single objective. Finally, the empirical evaluation across multiple real-world-inspired extremist network structures demonstrates the flexibility of the framework and highlights how network topology, resource constraints, and adversarial strategy interactions jointly influence disruption outcomes.

Despite these strengths, several limitations should be acknowledged. First, the proposed framework is designed as a stylized simulation environment rather than a full causal model of radicalization processes. Complex phenomena such as radicalization, recruitment, and de-radicalization are represented through simplified probabilistic transition mechanisms, which may not capture all real-world behavioral and sociopolitical factors. Second, the empirical evaluation is based on networks derived from specific datasets (e.g., PIRUS and SoNAR), which may introduce domain-specific biases and limit generalizability to other types of criminal or illicit networks. Differences in data availability, reporting practices, and network structure across domains may affect the transferability of learned strategies. Third, the current action space is limited to node-level interventions, whereas real-world counter-crime operations often involve more complex actions, including multi-target interventions, edge-level disruption, surveillance, and resource allocation strategies. Extending the framework to more expressive and combinatorial action spaces would improve realism but also increase computational complexity. Fourth, the learning framework assumes a parameterized transition model governing influence spread and recovery dynamics. While this enables tractable simulation and controlled experimentation, it may not fully capture heterogeneous or context-dependent mechanisms observed in real-world settings, such as delayed effects, external shocks, or multi-step persuasion processes. Finally, although the framework incorporates explainability mechanisms, the evaluation of explanation quality remains limited to internal consistency and feature alignment metrics. Future work could incorporate human-in-the-loop validation or user studies to assess the practical usefulness and interpretability of generated explanations in operational settings.

Overall, the proposed framework should be interpreted as a decision-support and exploratory modeling tool for studying adaptive disruption strategies under uncertainty. Future work can extend this approach by incorporating richer data sources, more realistic transition dynamics, and enhanced evaluation protocols to improve robustness and applicability in real-world.

## Methods

This section formulates the problem and describes a methodology for intervening in the spread of radicalization in terrorist networks based on MARL using non-zero-sum rewards and explainable competitive adversarial agents.

### Dataset description

This study utilizes the Global Terrorism Database (GTD), curated by the START consortium at the University of Maryland. Spanning over 200,000 terrorist incidents from 1970 to 2020, GTD is the most comprehensive open-access dataset of its kind^38^. PIRUS (Profiles of Individual Radicalization in the United States) is provided as an Excel dataset (PIRUS_V4.xlsx) and is subject to the PIRUS EULA (PIRUS_EULA_March2023.pdf). It contains profiles of individuals in the United States who radicalized to the point of committing ideologically motivated terrorist acts, spanning multiple decades. PIRUS includes demographic variables (e.g., age, gender, education, employment, social stratum), radicalization indicators (radical beliefs/behaviors, roles, and group membership), behavioral and activity variables (e.g., social media frequency, online radicalization pathway, recruitment/connection activity, violence indicator, terrorist severity), and psychological/vulnerability and temporal variables (e.g., psychological indicators, radicalization duration, year of exposure). Individuals may have up to four recorded group affiliations (Terrorist_Group_Name1–4), enabling multi-affiliation representation.

SoNAR provides undirected graph structure via node and edge files (SoNAR_Nodes.xlsx, SoNAR_Edges.xlsx). After standard filtering, the SoNAR graph contains 643+ connected nodes and 1,138+ edges. Nodes include Subject_ID (linkable to PIRUS), ideology, decade, and an In_PIRUS flag indicating the availability of a PIRUS profile. Edges represent observed relationships between individuals (Subject_A, Subject_B). The datasets are integrated using Subject_ID, mapping PIRUS attributes onto SoNAR nodes. Integration coverage varies by group, with connectivity ratios reported in the range 0.147–0.849 (group-dependent).

For group-level analysis, we focus on ten prominent extremist groups with sufficient profile availability and network feasibility: ISIL (248 profiles), Ku Klux Klan (172), Sovereign Citizens (146), QAnon (130), al-Qaeda core (110), Proud Boys (107), Christian Identity Movement (96), Boogaloo Movement (76), Three Percenters (69), and al-Shabaab (59). Group-specific subnetworks exhibit substantial heterogeneity in structure (density 0.026–0.135; average clustering 0.239–0.776; average degree 1.71–6.68; connected components 4–33), providing a diverse testbed for learning strategic disruption and intervention analysis.

#### Data limitations and potential biases

The data are subject to several known biases. First, selection bias arises because PIRUS predominantly captures individuals who were arrested, prosecuted, or publicly identified, likely underrepresenting less visible radicalization trajectories. Media and documentation bias may lead to overrepresentation and richer annotation for high-profile cases compared with low-profile cases. Temporal bias affects older cases, which may be less complete and often lack modern digital footprint indicators; apparent trends may be confounded by changes in reporting and collection practices over time. Reporting and classification bias may occur due to jurisdictional variation in law-enforcement documentation and because group affiliations can be noisy (multi-membership, evolving labels, and misclassification).

Incompleteness and measurement limitations are also important. Variable completeness differs by category: demographic variables are generally high completeness ($>90\%$), behavioral variables moderate (70–85%), psychological variables lower (60–75%), and temporal variables variable (50–90%). Some fields may be derived from interviews or statements and are therefore potentially affected by self-report and social desirability bias, while observed legal outcomes may be more reliable but incomplete. Finally, SoNAR edges may not capture all relationship types and provide limited longitudinal evolution, so the network is best interpreted as a structured snapshot rather than a full temporal relational history. Given the sensitivity of the data, all usage must comply with the PIRUS EULA and relevant institutional ethics requirements; analyses are reported in aggregated form to reduce re-identification risk.

#### Exploratory data analysis

Exploratory analysis characterizes dataset coverage, structural heterogeneity, and feature behavior across groups. At the profile level, we summarize group composition (top groups listed above) and key attribute distributions (e.g., age and activity indicators) to contextualize heterogeneity across ideologies and cohorts. At the network level, we compute group-specific statistics (nodes, edges, density, average clustering coefficient, average degree, and number of connected components), revealing marked variation from sparse, fragmented structures to denser, highly clustered communities. Degree distributions are generally right-skewed, indicating a small set of highly connected actors alongside many low-degree individuals; this supports later analyses of hub- and bridge-focused disruption patterns. Community detection and component structure highlight modular organization and varying separability across groups. Finally, we examine distributions and associations among engineered attributes (risk, influence, vulnerability) and network position (centralities), and we summarize missingness patterns by variable category to assess potential confounding effects due to incomplete annotation.

#### Data preprocessing

We preprocess PIRUS and SoNAR to construct analysis-ready attributed graphs suitable for modeling and learning. The pipeline loads PIRUS profiles and SoNAR nodes/edges, validates schema and identifiers, links profiles to nodes via Subject_ID, and produces full-network, PIRUS-only, and group-specific subnetworks. Centrality measures and community features are computed to support downstream influence and intervention modeling.

- **Data Cleaning.** Cleaning enforces identifier integrity and valid graph structure. Records with missing critical identifiers (e.g., Subject_ID) are excluded. SoNAR edges with missing endpoints are removed, and duplicate edges are dropped. The In_PIRUS indicator is standardized to a Boolean representation and cross-dataset consistency checks are performed (e.g., PIRUS IDs missing from SoNAR and vice versa; mismatches with In_PIRUS). Missing values are handled contextually: categorical fields are mapped to explicit “Unknown”/“No affiliation” values where meaningful; numerical fields are imputed conservatively using group-level summaries (e.g., median for age) or default values when absence plausibly indicates non-occurrence. Outliers in demographic, behavioral, and network-derived measures are checked for plausibility and validated against network structure; extreme values may be winsorized (e.g., capped near the upper tail) when appropriate, with all decisions documented.
- **Data Transformation.** Transformation constructs derived features and alternative network views. For node-level attributes, we compute composite scores used in modeling: a **radicalization risk score** (0–1) from weighted indicators (e.g., radical behaviors, radical beliefs, social media frequency, group membership, and violence), and an **influence score** combining network position (degree, betweenness, eigenvector centrality) with activity signals. We derive intervention-relevant variables including vulnerability and priority-style scores (balancing spread risk and susceptibility), as well as temporal variables (e.g., radicalization speed via inverse duration, time since exposure) when available. Network construction supports three views: (i) the full SoNAR network, (ii) a PIRUS-only subnetwork retaining nodes with In_PIRUS profiles, and (iii) group-specific subnetworks filtered by PIRUS group affiliations (Terrorist_Group_Name1–4) and induced on SoNAR.
- **Data Normalization.** Continuous engineered features and composite scores are primarily scaled using min–max normalization to the range [0, 1] to harmonize heterogeneous variables and stabilize learning-based components. When required (e.g., for comparability across groups with differing scale), network centrality measures are additionally standardized (z-scores). Binary influence states remain unscaled. Features with zero variance are detected and removed or assigned safe defaults to avoid numerical instability (e.g., division-by-zero during normalization). Where network-size effects could confound comparisons, structure-derived quantities are normalized by network size.

### Network construction

We construct terrorist networks using a multi-stage pipeline based on PIRUS and SoNAR datasets. Group affiliations are extracted across four PIRUS columns ($Terrorist\_Group\_Name1$ to *Name*4) to account for multi-affiliated individuals. Groups are scored on five weighted criteria–size, connectivity, edge count, density, and clustering–retaining only those with $\ge 10$ individuals and connected nodes. The top-9 groups selected include ISIL, Ku Klux Klan, Sovereign Citizens, QAnon, Proud Boys, Christian Identity Movement, Boogaloo Movement, Three Percenters, and al-Shabaab, based on structural robustness and real-world relevance. Networks are built by integrating 3,203 PIRUS profiles with 4,917 SoNAR nodes and 13,829 edges, filtered for group-specific relevance. Validation includes removal of NaN (not existed) edges, consistency checks, and enforcement of valid connections. The resulting structures reflect real-world fragmentation and covert behavior, with low density (0.0127) and high component count.

Each node is enriched with multidimensional features: a Radicalization Risk Score (based on ideology, group activity, and online behavior); an Influence Score (combining centrality measures and behavioral signals); and a Vulnerability and Resource Profile (covering psychological susceptibility, isolation, and estimated intervention cost). These attributes support explainable and targeted disruption strategies. The pipeline includes structural validations and logging to confirm that sparse connectivity stems from operational compartmentalization, not data quality. These validated networks provide a strong foundation for simulating radicalization dynamics and optimizing multi-agent counter-terrorism interventions.

### Evaluation metrics

We evaluate the performance of our MARL-based network disruption framework along three dimensions: intervention effectiveness, strategy robustness, and explainability.

Intervention effectiveness is measured by tracking changes in overall network integrity, fragmentation, and the size of the largest connected component following defender interventions. The network integrity at time step *t* is defined as

1 $$\begin{aligned} \begin{aligned} m_t^{\text {int}} = 1 - \frac{|v_{\text {inf}}|}{|V|} \end{aligned} \end{aligned}$$

where |*V*| is the total number of nodes in the network and $|v_{\text {inf}}|$ is the number of nodes currently radicalized or influenced by the attacker. This metric quantifies the proportion of the network that remains unaffected by radical ideology, with higher values indicating more successful defensive operations. To capture structural degradation, we compute the fragmentation increase as the change in the number of connected components, with larger increases reflecting greater disconnection of the network. Additionally, the reduction in the largest component size is measured as the difference in node count between the pre- and post-intervention largest connected component, serving as an indicator of core structural collapse.

To assess the strategic quality of agent’s behavior, we use two metrics: action diversity and strategic adaptation. Action diversity is measured using the Shannon entropy over the agent’s action distribution across time, capturing the balance between exploration and exploitation^39^. Higher entropy values suggest a richer and more context-sensitive action space. The strategic adaptation score evaluates how well agents adjust their policies in response to evolving attacker behavior, operationalized as a reward-weighted divergence between the agent’s past and current action distributions under similar network conditions. This reflects the agent’s learning capacity and ability to generalize strategic responses.

Finally, we incorporate explainability metrics to ensure the interpretability of agent decisions, which is crucial for real-world counter-terrorism applications. Explanation is assessed by comparing the alignment between the model’s action and its corresponding natural language explanation, ensuring that key node attributes (e.g., high centrality or radicalization risk) cited in the rationale are indeed influential in the decision-making process. Counterfactual sensitivity is examined by perturbing individual node features–such as influence or vulnerability scores–and observing whether the agent’s action changes accordingly^40^. This helps identify decision boundaries and assess model robustness. In addition, we evaluate strategy abstraction coverage by clustering action sequences across episodes into macro-strategies such as “hub neutralization” and “bridge isolation” and measuring their representation frequency^41,42^. High coverage of interpretable, recurring strategies signals not only coherent behavior but also actionable insights for analysts. Together, these metrics enable a comprehensive evaluation of the performance, blending structural impact, behavioral intelligence, and interpretability for robust and explainable terrorist network disruption

**Fig. 7:**
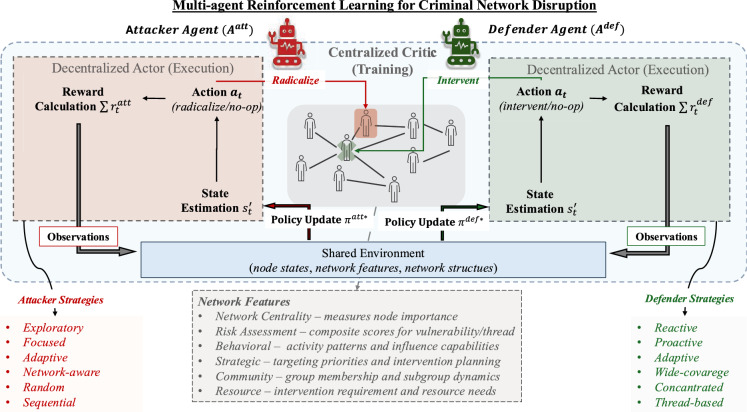
Overview of the proposed MARL framework for network disruption. The diagram illustrates the interaction between attacker and defender agents within a shared network environment, including observations, actions, reward signals, and policy updates. To navigate the figure, follow the flow from observations to actions and rewards for each agent, highlighting the feedback loop through which policies are updated. The key message is that disruption is modeled as a sequential, adversarial learning process in which both agents adapt over time under partial observability, enabling the emergence of strategic behavior and interpretable decision patterns.

### Framework

Figure 7 illustrates the MARL framework for terrorist network disruption task as a sequential game between two intelligent agents interacting within a dynamic graph environment. Each agent receives partial observations of the network, including topological features, node centrality metrics, and behavioral signals. Each agent learns a stochastic node-selection policy from experience rather than following a fixed ranking rule. The current network state is encoded as a feature vector, passed through a neural policy/value network, and used to produce both a probability distribution over candidate nodes and a bootstrap estimate of future return. Training is performed from replayed transitions using temporal-difference targets and gradient-based optimization. This allows node selection to depend on expected long-term consequences of actions rather than solely on instantaneous influence or centrality scores.

Partial Observability and State Representation. The assumption of partial observability reflects the realities of counter-terrorism and intelligence operations, where complete knowledge of network structure and individual behavior is rarely available. In practice, information about relationships, influence pathways, and individual roles is often incomplete, noisy, or delayed, resulting in uncertainty about the true underlying network state. In this work, partial observability is modeled through a feature-based representation of the network rather than direct access to the full graph structure. Specifically, each agent observes a set of aggregated and node-level descriptors, including centrality measures, behavioral indicators, and risk-related attributes. This design choice serves two purposes. First, it provides a tractable and stable representation for learning, avoiding the complexity associated with variable-size graph observations. Second, it supports the interpretability objectives of the framework, as these features are directly aligned with the explanation mechanisms used to justify agent decisions. While richer observation models based on partial subgraphs or local neighborhoods could provide a more detailed representation of network structure, they would introduce additional complexity in both learning and interpretation. We therefore adopt a feature-based abstraction as a principled trade-off between realism, tractability, and explainability, leaving more expressive observation models as an avenue for future work.

The attacker agent employs one of several strategies to expand its influence across the network by radicalizing nodes. Meanwhile, the defender agent implements intervention policies to minimize the spread of radicalization. Both agents receive distinct reward signals derived from the network’s evolving state, including influence spread, connectivity changes, and resource efficiency. Policy updates are performed independently based on reinforcement learning objectives. This setup reflects real-world counter-crime dynamics by capturing both strategic behavior and the structural complexity of covert networks.

Let the network at time *t* be represented by the attributed graph $\mathscr {G}_t = (V_t, E_t, X_t, A_t)$, where $V_t$ denotes the set of nodes corresponding to terrorist actors, $E_t \subseteq V_t \times V_t$ is the set of edges representing their interactions, $X_t \in \mathbb {R}^{|V_t| \times d}$ represents node-level features such as role, lethality, or centrality, and $A_t \in \mathbb {R}^{|E_t| \times k}$ encodes edge-level properties such as event co-participation or communication intensity. The objective of the disruption problem is to identify a subset of nodes or edges $S_t \subseteq V_t \cup E_t$ to remove at each time step, subject to a fixed intervention budget $|S_t| \le B$. The optimal strategy aims to maximize a disruption objective $\mathscr {L}$ defined over the modified network structure and defined as $\max _{S_t \subseteq V_t \cup E_t,\ |S_t| \le B} \mathscr {L}(\mathscr {G}_t \setminus S_t)$.

Various formulations of $\mathscr {L}$ are used depending on the strategic goals. For instance, network fragmentation can be expressed as minimizing the size of the largest connected component $\mathscr {L}_{\text {frag}} = - \left| \text {LCC}(\mathscr {G}_t \setminus S_t) \right|$. Alternatively, influence minimization models aim to reduce the expected spread $\sigma$ under a diffusion model (e.g., Independent Cascade) $\mathscr {L}_{\text {influence}} = - \sigma (\mathscr {G}_t \setminus S_t)$. For role-based targeting, the objective may focus on cumulative disruption of high-utility nodes $\mathscr {L}_{\text {utility}} = - \sum _{v \in V_t \setminus S_t} f(v)$ where *f*(*v*) denotes the strategic importance or function score of node *v*.

To capture the strategic dynamics between attackers and defenders over time, we formalize the task as a partially observable stochastic game with two learning agents: an attacker $\mathscr {A}^{\text {att}}$ and a defender $\mathscr {A}^{\text {def}}$. The environment is described by $\mathscr {M} = \langle \mathscr {S}, \mathscr {A}^{\text {att}}, \mathscr {A}^{\text {def}}, \mathscr {T}, \mathscr {O}, R^{\text {att}}, R^{\text {def}}, \gamma \rangle$ where $\mathscr {S}$ denotes the (partially observable) state of the network, $\mathscr {T}$ is the transition function modeling the evolution of the network, $\mathscr {O}$ is the observation model, and $R^{\text {att}}$ and $R^{\text {def}}$ are the reward functions for the attacker and defender, respectively. The discount factor $\gamma \in [0,1]$ encodes the importance of future rewards. Each agent learns a policy $\pi$ that maximizes its expected discounted return:

2 $$\begin{aligned} \pi ^* = \arg \max _{\pi } \mathbb {E} \left[ \sum _{t=0}^{T} \gamma ^t R_t \right] \end{aligned}$$

In this setting, the attacker aims to preserve influence, propagate radicalization, and maintain structural robustness, while the defender seeks to fragment the network, suppress high-impact actors, and limit the possibility of cascade effects. By incorporating graph-theoretic metrics, behavioral features, and temporal dynamics into the agent’s observation and reward structures, the model enables the simulation and learning of adaptive disruption policies in evolving, adversarial environments.

#### Environment and state transitions

The environment is governed by two competing agents with opposing objectives. The *attacker agent*, denoted by policy $\pi ^{att}: \mathscr {S} \rightarrow \Delta (\mathscr {A}^{att})$, models the decision-making process of a terrorist organization, aiming to maximize the spread of radicalization through strategic influence of nodes. The *defender agent*, denoted by policy $\pi ^{def}: \mathscr {S} \rightarrow \Delta (\mathscr {A}^{def})$, represents counter-crime operations that seek to minimize network radicalization by applying targeted interventions such as deradicalization or prevention. Both agents operate over a shared discrete action space defined as $\mathscr {A}^{att} = \mathscr {A}^{def} = \{0, 1, 2, \ldots , |V|-1\} \cup \{\text {no-op}\}$ where each action corresponds to selecting an individual node in the network for intervention, and the special action *no-op*, meaning *do not select*, allows an agent to abstain from acting at that timestep. The goal of each agent is to learn a policy that maximizes its expected discounted reward. The attacker seeks:

3 $$\begin{aligned} \begin{aligned} \pi ^{att*} = \arg \max _{\pi ^{att}} \mathbb {E}_{\tau \sim \pi ^{att}, \pi ^{def}}\left[ \sum _{t=0}^{T} \gamma ^t r_t^{att}(s_t, a_t^{att}, a_t^{def})\right] \end{aligned} \end{aligned}$$

while the defender optimizes:

4 $$\begin{aligned} \begin{aligned} \pi ^{def*} = \arg \max _{\pi ^{def}} \mathbb {E}_{\tau \sim \pi ^{att}, \pi ^{def}}\left[ \sum _{t=0}^{T} \gamma ^t r_t^{def}(s_t, a_t^{att}, a_t^{def})\right] \end{aligned} \end{aligned}$$

where $\tau$ denotes a joint trajectory, *T* is the episode horizon, $\gamma$ is the discount factor, and $r_t^{att}, r_t^{def}$ are the respective attacker and defender reward functions. This setup compose a non-zero-sum Markov game, where $r_t^{att} + r_t^{def} \ne 0$. This formulation captures the realistic dynamics of counter-crime operations, where both successful influence and partial defensive gains may coexist. Defensive success does not necessarily negate all attacker progress, and vice versa.

The environment implements state transitions that model realistic influence spread and recovery dynamics within terrorist networks. When we consider the state transition in terrorist network, we decomposed into two independent process: *influence spread* and *recovery* for attacker’s and defender’s effect, respectively. Influence spread defined as when an attacker targets individual *v* at time *t*, the probability of successfull influence is $P_{\textit{inf}}(s_{t+1}|v, s_t, a_t^{att}, a_t^{def}) = p_{\text {base}} \cdot \phi _{\text {imp}}(v) \cdot \phi _{\text {def}}(v, a_t^{def})$ where:

5 $$\begin{aligned} \phi _{\text {imp}}(v)&= 1 + \beta \cdot \textit{importance}(v) \end{aligned}$$

6 $$\begin{aligned} \phi _{\text {def}}(v, a_t^{def})&= {\left\{ \begin{array}{ll} 1 - \delta & \text {if } a_t^{def} = v \\ 1 & \text {otherwise} \end{array}\right. } \end{aligned}$$

with base probability $p_{\text {base}}$ representing the fundamental difficulty of radicalization, importance scaling $\beta$ represents that the nodes has high importance is easier to radicalize, and defense effectiveness $\delta$ represents the defence effectiveness. When a defender targets influenced individual *v*, recovery probability defined as $P_{\text {rec}}(s_{t+1}|v, s_t, a_t^{def}) = p_{\text {rec}} \cdot \psi _{\text {str}}(v) \cdot \psi _{\text {time}}(v)$ where:

7 $$\begin{aligned} \psi _{\text {str}}(v)&= 1 + \gamma \cdot \textit{importance}(v) \end{aligned}$$

8 $$\begin{aligned} \psi _{\text {time}}(v)&= 1 + \xi \cdot \min \bigg (0.5, \frac{\textit{time\_inf}(v)}{T_{\text {max}}}\bigg ) \end{aligned}$$

with base recovery probability $p_{\text {rec}}$, strategic bonus $\gamma$, and time factor $\xi$. Therefore, the joint transition probability defined as $P(s_{t+1} | s_t, a_t^{att}, a_t^{def}) = P_{\text {inf}}\big (s_{t+1} | s_t, a_t^{att}, a_t^{def}) \cdot P_{\text {rec}}(s_{t+1} | s_t, a_t^{def}\big ).$

State–Action Design and Temporal Dependency. At each time step, the environment state is represented by the current network configuration, including node attributes and connectivity structure. Actions correspond to selecting a node for intervention (e.g., influence, disruption, or stabilization), which modifies the state through changes in node status, influence propagation, and structural relationships. Importantly, the effect of an action is not limited to the immediate state but propagates over time through the network. Selecting a node can alter future influence dynamics, modify connectivity patterns, and affect the strategic behavior of the opposing agent. As a result, the value of an action depends on its long-term impact on network evolution rather than solely on instantaneous structural properties. This induces a sequential decision-making problem in which policies must account for delayed and cumulative effects. Consequently, selecting nodes based solely on static metrics such as centrality is generally suboptimal, as it ignores downstream consequences and adversarial adaptation. The proposed learning framework enables agents to discover strategies that balance immediate gains with long-term objectives under dynamic and adversarial conditions.

### Reward system design

Early and classical game-theoretic models–especially those developed for military conflict, security, and competitive economics–often assumed a strictly zero-sum reward structure, where one player’s gain is exactly the other’s loss, enforcing a zero-sum constraint of the form $r_t^{att} + r_t^{def} = 0$. While mathematically convenient, this approach fails to reflect the realistic dynamics of real-world counter-crime settings. In practice, both terrorist groups and counter-crime agencies can achieve simultaneous successes. For example, a radical group may influence certain individuals even as law enforcement prevents attacks elsewhere. This decoupling of outcomes across time and space invalidates the immediate antagonism imposed by zero-sum formulations. Moreover, enforcing perfectly negative reward correlation ($-1.0$) can lead to training instabilities, limiting policy diversity and inhibiting strategic exploration.

To address these limitations, we adopt a non-zero-sum Markov game structure where $r_t^{att} + r_t^{def} \ne 0$. This allows both agents to receive positive rewards simultaneously, enabling the modeling of competing but independently measurable objectives. Our formulation preserves adversarial tension while allowing for more realistic, asynchronous reward feedback, better reflecting the distributed and multifaceted nature of real-world counter-terrorist operations. It also promotes stable learning, richer behavioral diversity, and more adaptive multi-agent interactions.

**Attacker Rewards.**

The attacker’s reward function at time step *t*, denoted as $r_t^{att}$, and defined as $\sum r^{att}_{i}$ where *i* is in {base, strategic, cascade, disruption, control}. The *base reward* is formulated as $r_{\text {base}}^{att} = w_{\text {base}} \cdot [\textit{inf\_success}(a_t^{att})]$ providing direct incentive for each successful radicalization attempt, thus encouraging active propagation rather than passivity. The *strategic reward* is given by $r_{\text {str}}^{att} = w_{\text {str}} \cdot \textit{imp}(v_{a_t^{att}}) \cdot [\textit{inf\_success}(a_t^{att})],$ where $\textit{imp}(v)$ represents node importance, defined as a weighted combination of betweenness, eigenvector, and degree centralities. This term encourages the attacker to prioritize strategically significant nodes, enabling wider and more impactful influence spread. The *cascade reward* is expressed as $r_{\text {casc}}^{att} = w_{\text {casc}} \cdot |\textit{cascade\_nodes}_t|,$ where $\textit{cascade\_nodes}_t = \{u \in N(v_{a_t^{att}}): \textit{newly\_inf}(u)\}$. This captures the benefit from secondary influences via neighborhood contagion, fostering strategies that exploit network topology for chain reactions. The *disruption reward* is formulated as $r_{\text {disrup}}^{att} = w_{\text {disrup}} \cdot (1 - \textit{integrity}_t),$ with $\textit{integrity}_t = 1 - \frac{|\textit{inf\_nodes}_t|}{|V|}.$ This term incentivizes broader network degradation, aligning attacker objectives with systemic destabilization rather than only local success. Finally, the *control reward* is defined as

9 $$\begin{aligned} r_{\text {control}}^{att} = {\left\{ \begin{array}{ll} \textit{inf\_ratio}_t - 0.2, & \text {if } \textit{inf\_ratio}_t> 0.2, \\ 0, & \text {otherwise}, \end{array}\right. } \end{aligned}$$

where $\textit{inf\_ratio}_t = 1-\textit{integrity}_t$ This term motivates achieving a significant level of total influence, encouraging long-term planning and global dominance.

Collectively, these reward components ensure the attacker balances immediate influence successes with strategic and large-scale objectives, emulating realistic and adaptive adversarial behaviors.

Defender Rewards. The defender’s reward function at time *t*, denoted as $r_t^{def}$, and defined as $\sum r^{def}_{i}$ where *i* is in {rec, protection, integrity, improvement, prevention}. The *recovery success reward* is defined as

10 $$\begin{aligned} \begin{aligned} r_{\text {rec}}^{def} =&w_{\text {rec}}\cdot [\textit{rec\_success}(a_t^{def})] + w_{\text {str\_recovery}}\cdot \textit{imp}(v_{a_t^{def}})\cdot [\textit{rec\_success}(a_t^{def})]. \end{aligned} \end{aligned}$$

This incentivizes successful deradicalization, particularly targeting high-importance nodes, reflecting targeted counter-radicalization strategies. The *protection reward* is given by $r_{\text {prot}}^{def} = w_{\text {prot}}\cdot [\textit{prev\_success}(a_t^{def})].$ This rewards the defender for actively blocking attacker influence attempts, emphasizing preemptive defense. The *network integrity reward* is formulated as $r_{\text {integ}}^{def} = w_{\text {integ}}\cdot \textit{integ}_t.$ This encourages maintaining overall network health, focusing on global stability rather than isolated interventions. The *improvement bonus* is defined as $r_{\text {imp}}^{def} = w_{\text {imp}}\cdot \max (0, \textit{integ}_t - \textit{integ}_{t-1})$ and it promotes incremental positive changes, fostering continuous improvement and proactive resilience-building. Finally, the *high integrity bonus* is given by

11 $$\begin{aligned} r_{\text {prev}}^{def} = {\left\{ \begin{array}{ll} w_{\text {high}}, & \text {if } \textit{integ}_t> 0.8, \\ \frac{w_{\text {high}}}{2}, & \text {if } 0.6 < \textit{integ}_t \le 0.8, \\ 0, & \text {otherwise}. \end{array}\right. } \end{aligned}$$

This encourages maintaining high integrity levels over time, rewarding sustained success and discouraging complacency.

Together, these components provide a robust, multi-layered framework that aligns the defender’s incentives with both immediate protective actions and long-term network resilience, promoting strategic, adaptive defense policies.

### Policy representation and training objective

At each time step, the environment state is encoded as a fixed-dimensional vector $s \in \mathbb {R}^{d}$ comprising node indicators, node-level attributes, and normalized structural descriptors of the current network configuration, such as degree-based and local clustering summaries. Although the underlying environment is graph-structured, the agents do not operate directly on raw adjacency matrices or explicit partial subgraphs. Instead, learning is performed on this feature-based state representation, which provides a tractable and interpretable abstraction of the evolving network.

For each agent $i \in \{\textrm{att}, \textrm{def}\}$, the action space is the set of node indices $\mathscr {A} = \{1,\dots ,N\},$ where selecting action $a \in \mathscr {A}$ corresponds to intervening on the associated node. Each agent uses a parameterized stochastic policy $\pi _{\theta _i}(a \mid s)$ together with a state-value function $V_{\phi _i}(s)$. In our implementation, both outputs are produced by a shared neural network backbone: one output head produces logits over nodes, which are transformed by a softmax to obtain $\pi _{\theta _i}(a \mid s)$, and a second output head produces a scalar estimate of the value function $V_{\phi _i}(s)$.

Transitions of the form $(s_t, a_t, r_t, s_{t+1}, d_t)$ are stored in an agent-specific replay buffer $\mathscr {D}_i$, where $d_t \in \{0,1\}$ indicates episode termination. During training, minibatches $\mathscr {B}$ of size *B* are sampled uniformly from $\mathscr {D}_i$. For each sampled transition, we define the temporal-difference bootstrap target as $y_t = r_t + \gamma (1-d_t) V_{\phi _i}(s_{t+1}),$ where $\gamma \in (0,1)$ is the discount factor. In the primary training path, gradients are stopped through the bootstrap target, following the standard semi-gradient temporal-difference setting. The policy parameters are updated by minimizing the objective

12 $$\begin{aligned} \mathscr {L}_{\pi }^{(i)} = -\frac{1}{B} \sum _{(s_t,a_t,r_t,s_{t+1},d_t)\in \mathscr {B}} \log \!\bigl (\pi _{\theta _i}(a_t \mid s_t) + \epsilon _{\textrm{num}}\bigr )\, y_t, \end{aligned}$$

where $\epsilon _{\textrm{num}}> 0$ is a small numerical constant. Gradients of $\mathscr {L}_{\pi }^{(i)}$ with respect to $\theta _i$ are computed by backpropagation, clipped to control instability, and optimized by using Adam.

Importantly, this learning procedure is not tabular Q-learning. No $|S|\times |\mathscr {A}|$ table is maintained, and no explicit action-value function *Q*(*s*, *a*) is stored. Instead, the action distribution is learned directly through the policy network $\pi _{\theta _i}(\cdot \mid s)$, while the value head provides a bootstrap estimate used for temporal-difference learning. Graph-derived quantities such as centrality or influence may appear as components of the state representation or the reward function, but they do not themselves define the policy. Consequently, node selection is not performed by simply choosing the most influential node according to a fixed heuristic; rather, it is determined by a learned stochastic policy conditioned on the evolving state of the environment.

A common actor–critic presentation would replace the target weighting in Eq. (12) with an advantage term $\hat{A}_t = y_t - V_{\phi _i}(s_t)$ and a separate value regression loss, for example $\beta \bigl (V_{\phi _i}(s_t)-y_t\bigr )^2$. Our primary implementation uses the update in Eq. (12), which preserves the same core principle of jointly learning a policy and a value-based bootstrap signal.

### Problem complexity and challenges

The terrorist network disruption problem poses a range of computational and theoretical challenges. First, the curse of dimensionality arises due to the large number of individuals in the network and the high-dimensional feature space, resulting in an exponentially growing state space that makes exact solution methods intractable. Partial observability further complicates the problem, as real-world counter-crime operations often operate with incomplete information regarding network topology, individual behaviors, and ongoing activities, necessitating decision-making under uncertainty. The problem is also inherently non-stationary, since both attacker and defender agents learn and adapt over time, making the environment appear dynamic and unstable from each agent’s perspective. Moreover, the setting involves optimization, where defenders must simultaneously optimize for disruption effectiveness, operational efficiency, legal compliance, and the minimization of unintended consequences. Finally, temporal dependencies in the network’s evolution play a crucial role–decisions taken at a given moment can influence future states through cascading radicalization effects, adversarial adaptation, and broader community-level responses.

## Conclusion

This work presented an explainable, game-theoretic multi-agent reinforcement learning framework for studying terrorist network disruption as a sequential and adversarial decision-making problem. By modeling the interaction between attacker and defender agents over partially observable and evolving influence graphs, the proposed framework extends beyond static network interdiction approaches and enables the analysis of adaptive disruption dynamics under uncertainty.

The empirical evaluation across multiple real-world-inspired extremist network structures demonstrated that disruption effectiveness depends not only on resource availability, but also on the interaction between attacker and defender strategies and on the structural properties of the underlying network. The results showed that increasing intervention budgets generally improves disruption performance, although the magnitude and consistency of these gains vary substantially across groups. Furthermore, the observed strategy-dependent outcomes indicate that effective intervention policies must account for adversarial adaptation and network-specific characteristics rather than relying on fixed or purely centrality-driven targeting rules.

A central contribution of this study is the integration of explainability into the learning framework. By associating intervention decisions with interpretable rationales derived from structural and behavioral network indicators, the proposed approach provides analyst-facing transparency into how and why specific nodes are targeted over time. This moves beyond black-box optimization and supports the development of interpretable decision-support systems for intelligence-led policing and counter-crime analysis.

At the same time, the study highlights important challenges and opportunities for future research. The current framework adopts a stylized representation of influence propagation and adversarial interaction, and therefore should be interpreted as an exploratory simulation and decision-support model rather than a full causal representation of radicalization processes. Future work may extend the framework through richer observation models, combinatorial intervention strategies, graph-native policy architectures, more realistic temporal dynamics, and human-centered evaluation of explanation quality. Incorporating additional data modalities and stronger validation protocols would further improve realism, robustness, and operational relevance.

Overall, the findings suggest that explainable learning-based approaches provide a promising direction for analyzing adaptive intervention strategies in complex criminal and extremist networks. By combining sequential decision-making, adversarial interaction, and interpretable reasoning within a unified framework, this work contributes toward the broader development of transparent and adaptive AI systems for high-stakes networked environments.

## Data Availability

The authors confirm that the data that support the findings of this study are openly available in the Global Terrorism Database (GTD) and the sources of the data supporting the findings of this study are available within the article.
